# The Principles and Practice of Medicine

**Published:** 1843-11-11

**Authors:** 


*The Principles and Practice of Medicine.* By John Elliotson, M. D. Cantab., F. R. S., etc. etc. Edited by Nathanial Rogers, M. D., etc., and Alexander Cooper Lee. *First American, from the Second London Edition, Greatly Enlarged and Improved. With Notes and Additions.* By Thomas Stewardson, M. D., Physician to the Philadelphia Hospital. Philadelphia: Carey and Hart. 1844. 8vo. pp. 1046.

Dr. Elliotson tells us in the Introduction to this work, that although he toiled very hard, published book after book, edition after edition, and saw "paper after paper honoured with a place in the Transactions of the first Medical Society in Europe," still most of his contemporaries, and even his juniors, who worked less, passed by him. He was Physician, too, to a large Metropolitan Hospital, and had attended there, and gratuitously out of doors, above twenty thousand patients. But all in vain. "In 1828, my profession was no more lucrative to me than in 1818, and was as short of my actual expenses." At that time the London Lancet, a journal originally on the same plan as our own, published, occasionally, a lecture of the Doctor's, delivered at St. Thomas' Hospital. His practice, to use his own words, "at once doubled." "The following year it published the greater part as I delivered them, and my practice doubled again. Next season the Lancet published them all; the Medical Gazette followed its example; my practice doubled a third time." Tbis is a single history of a hundred similar cases; and the eagerness with which Clinical and other Lecturers seek the hebdomadals attests the fact.

The present bulky volume comprises these same lectures, grouped together, as the Editors inform us, in conformity with Dr. Elliotson's views, and arranged in parts, books, chapters, and sections. Had the Editors stopped here all had been well; but, on the first edition being rapidly exhausted, as we can readily conceive of, it was thought necessary to enlarge the work, and make numerous additions. Dr. Rogers, besides being a prince of bombast in style, is one of those insatiable borderers who levies black mail on all sides, and a very nice and curiously wrought mosaic he has made of it. But the intrinsic merits of the book were too great to be destroyed, even by so accomplished a bungler, and to the atrocities of the English editor, we have, as an offset, the judicious and clever additions of the American editor.

It is not within the scope of this journal to notice, in detail, so extensive a work as the present. We have long been familiar with the lectures of Dr. Elliotson. They are eminently practical, containing much that is sound, both in diagnosis and therapeutics, and the style of communication is commendable.

The numerous affections are treated of in the following order: After a chapter on General Pathology, in which the nature of disease, Nosology, Etiology, Semeiology, and General Treatment of Diseases, are discussed, we have, forming the first part, General Diseases. This division includes Inflammation, Hæmorrhage, Profluvia, Dropsy, Deficient Secretions, Changes of Structure, Transformation of Structure, and New Formations.

Part second treats of Universal Diseases, among which our authors rank Anemia, Chlorosis, Scurvy, and the Fevers. The third and last part, is devoted to the conderation of the Local Diseases. It is divided into seven books. I, Cutaneous Diseases. II, Diseases of the Nervous System. III, Diseases of the Respiratory Organs. IV, Diseases of the Heart. V, Diseases of the Chylopœitic Viscera. VI, Diseases of the Urinary Organs. VII, Diseases of the Fibrous Tissues.

In the present edition Dr. Stewardson has added an excellent chapter on Remittent Fever, which we should much like to notice more in detail, and one on Yellow Fever. Both are distinguished by the discriminating mind of that gentleman. Besides these we have some excellent remarks by him on Cholera Infantum, on Pneumonia, Delirium Tremens, Tubercular Meningitis,

Small Pox, Hæmoptysis, Continued Fever, &c. &c., all cleverly treated.

In conclusion we recommend this volume both to the student and practitioner, as, on the whole, one of the best compendiums on the Practice of Medicine which has yet been published.

